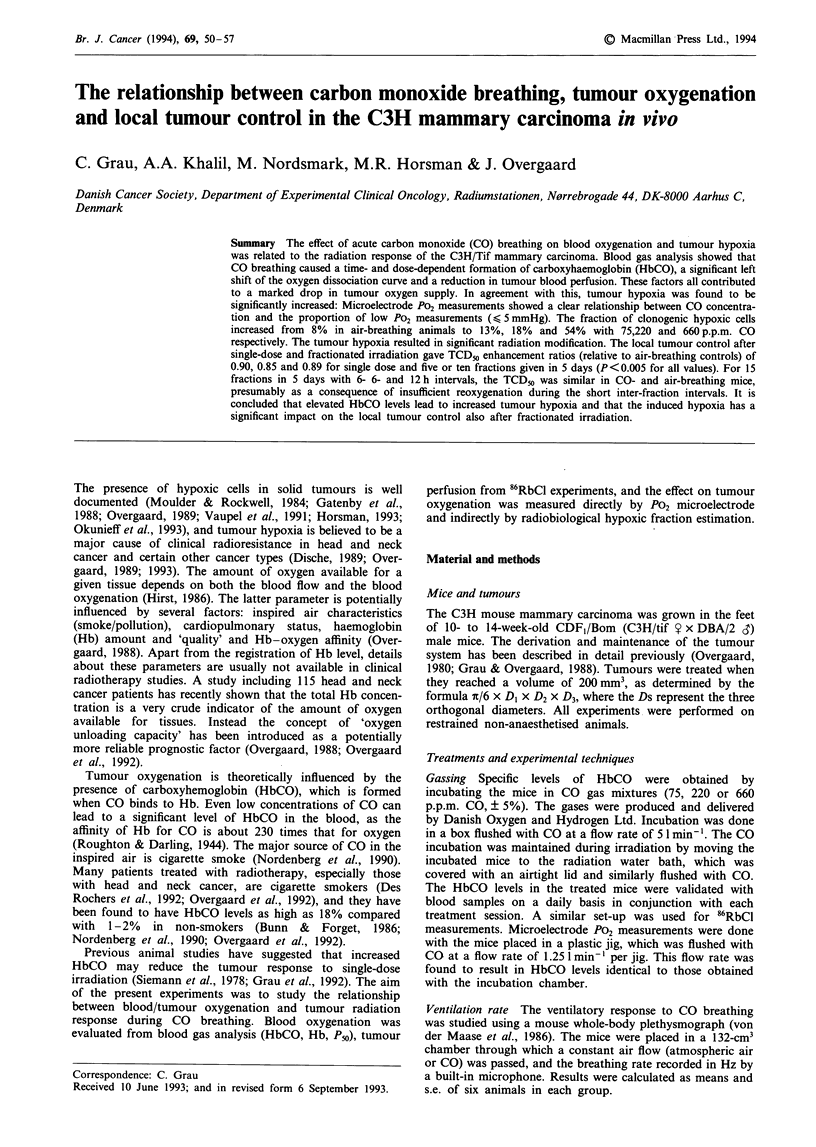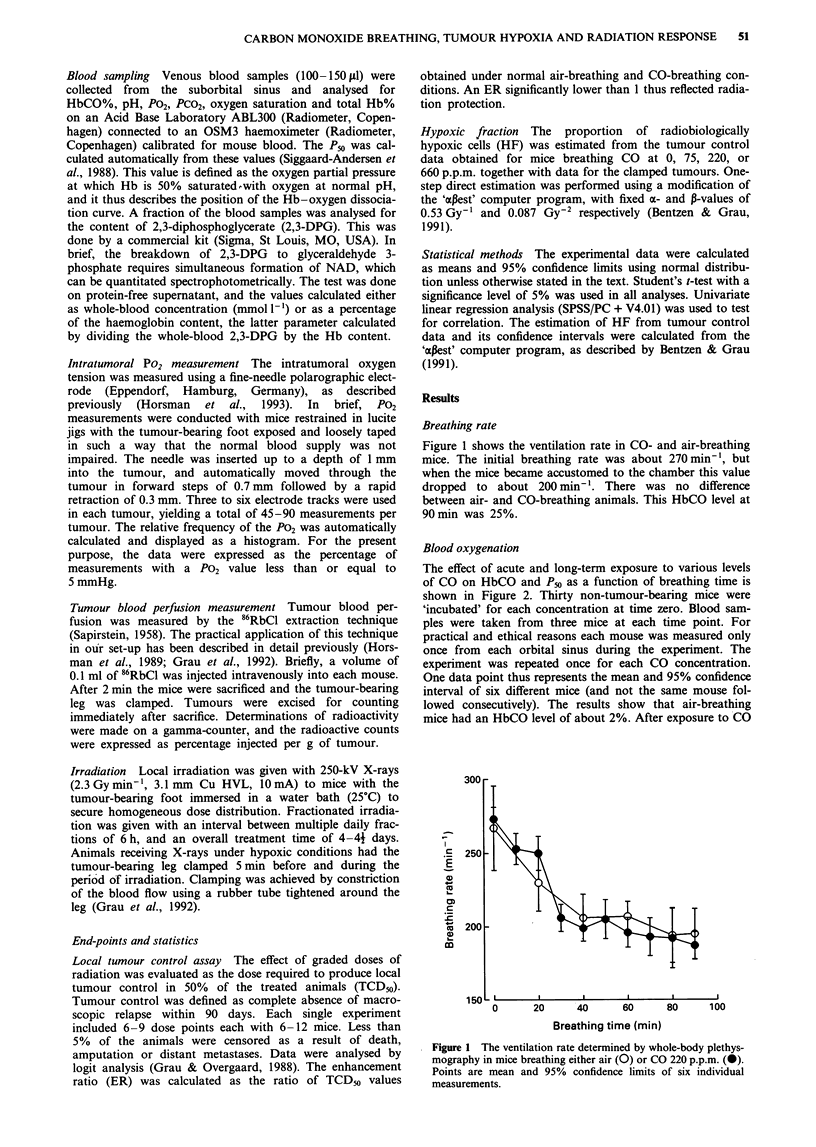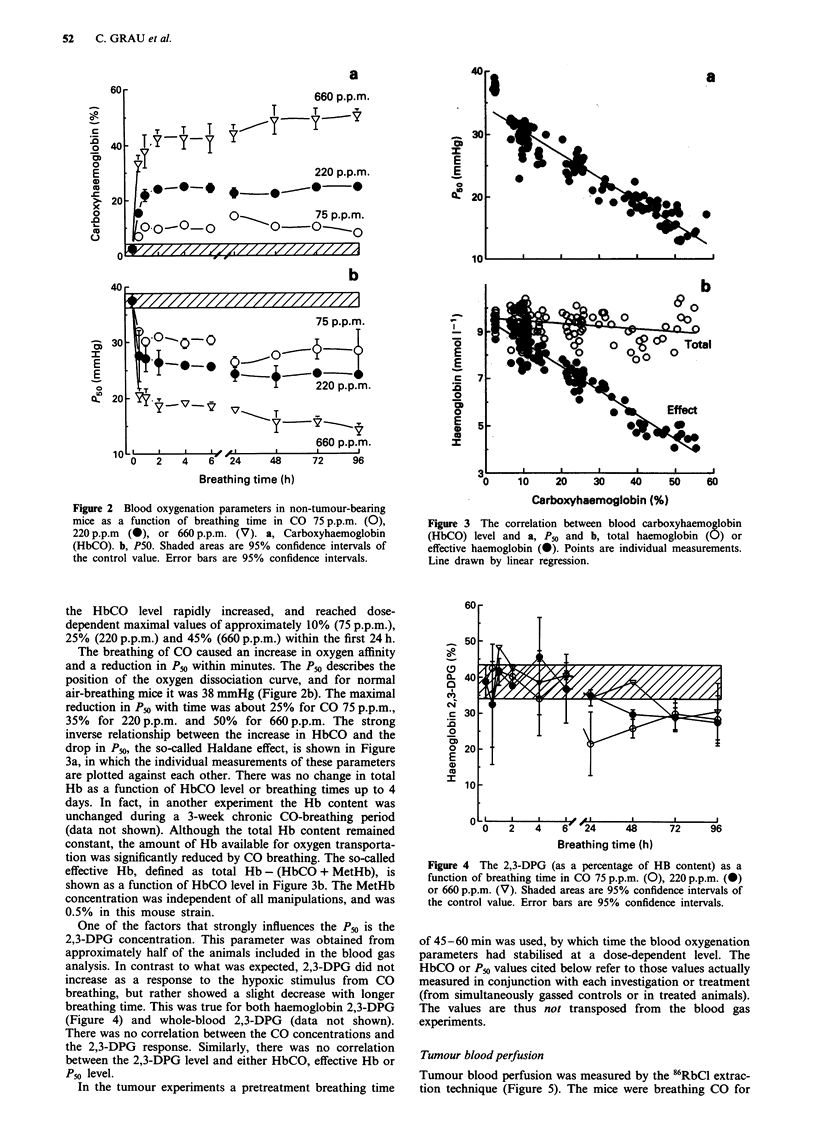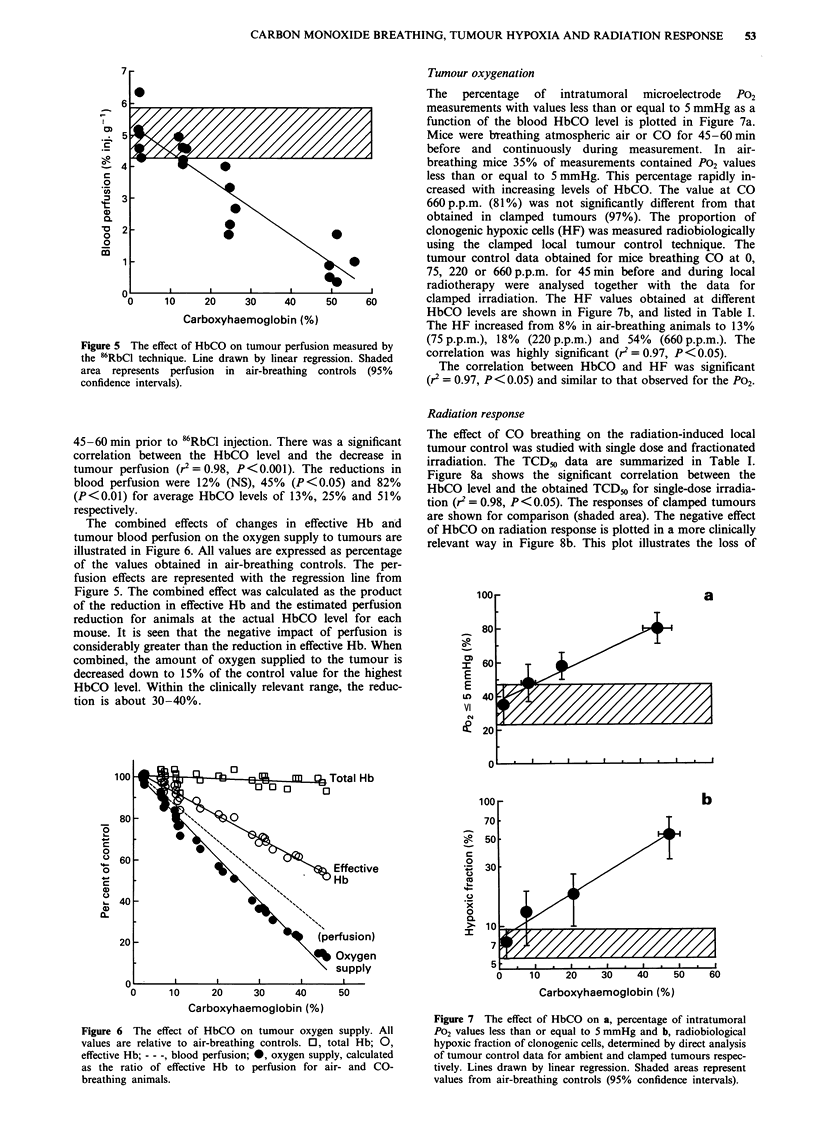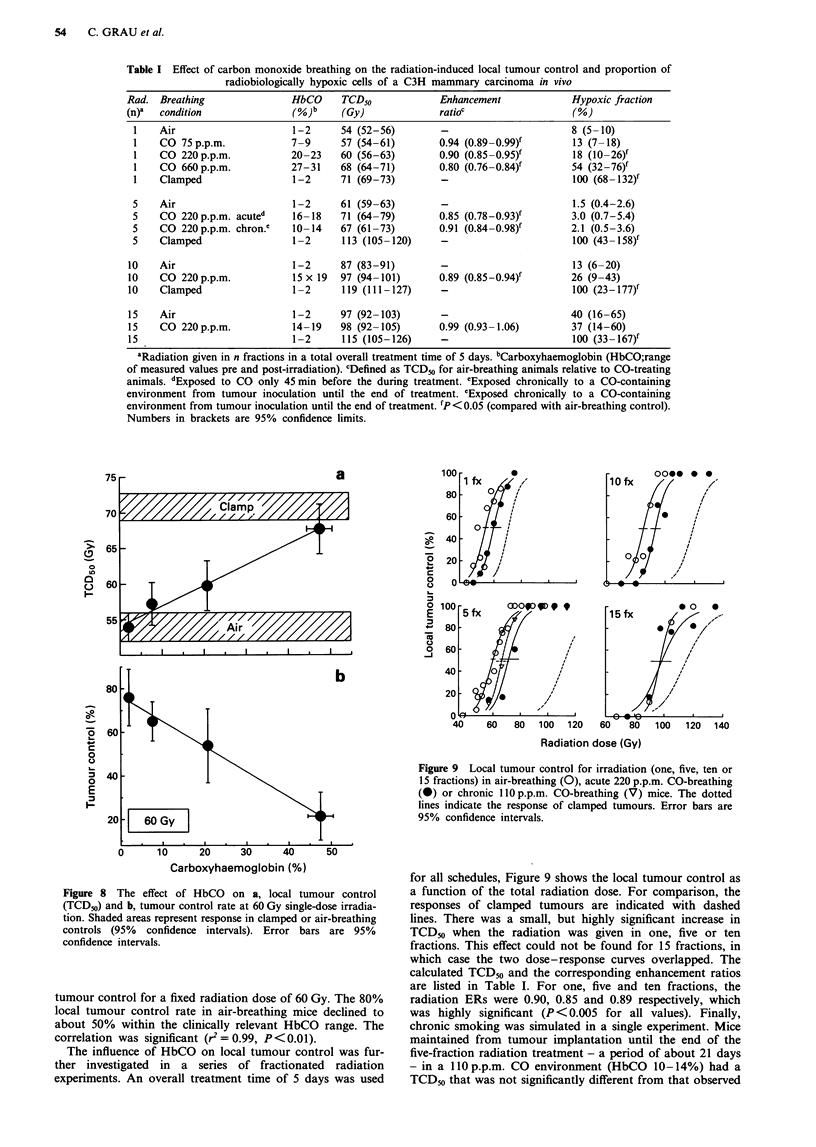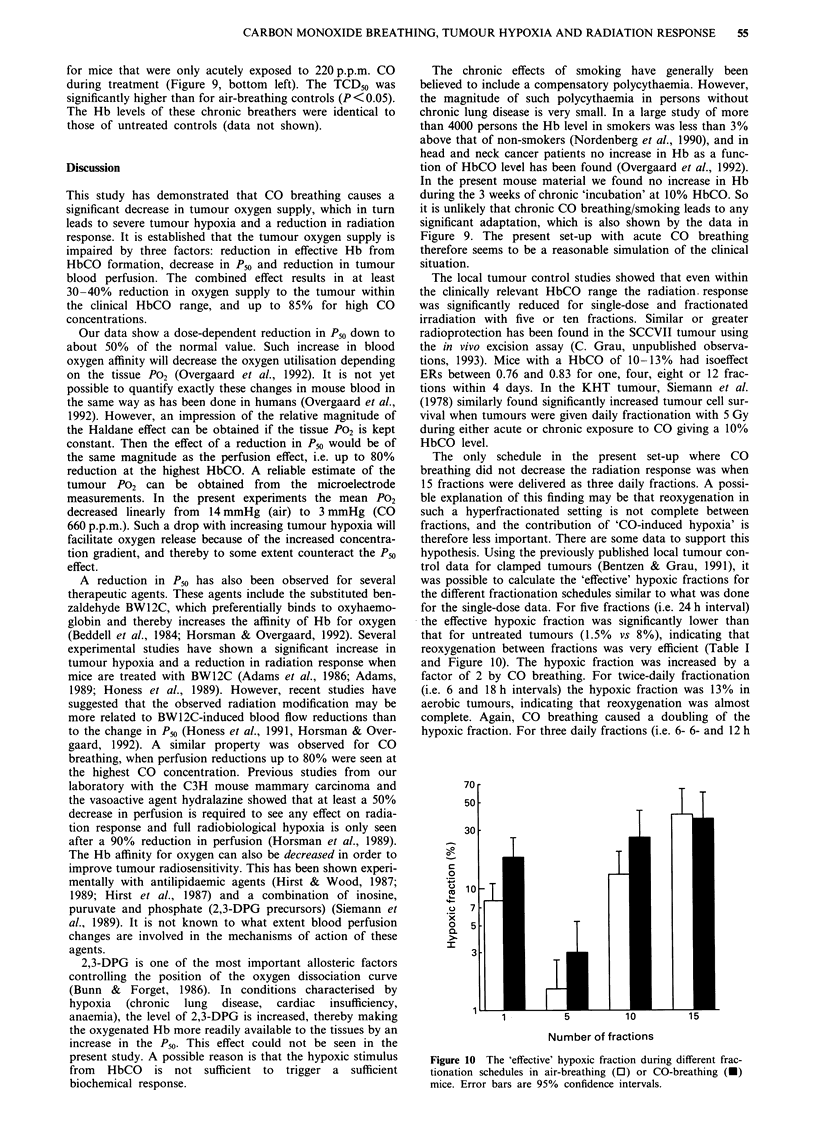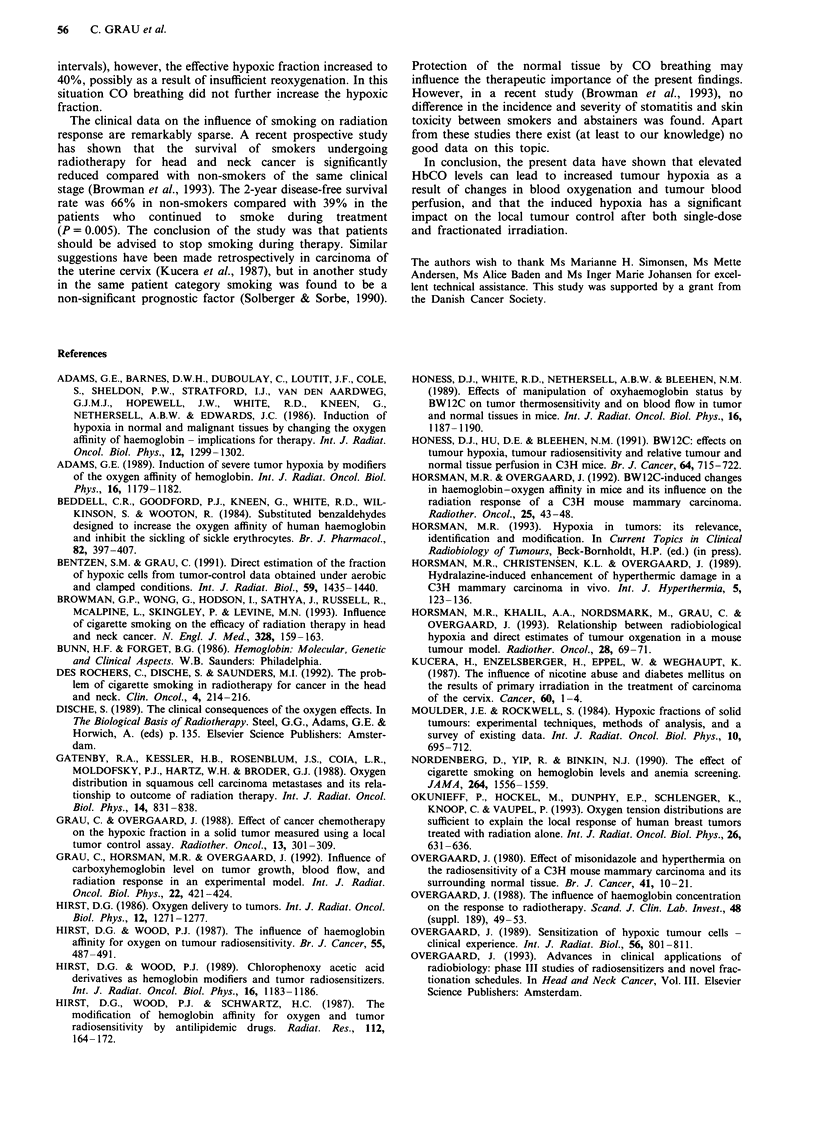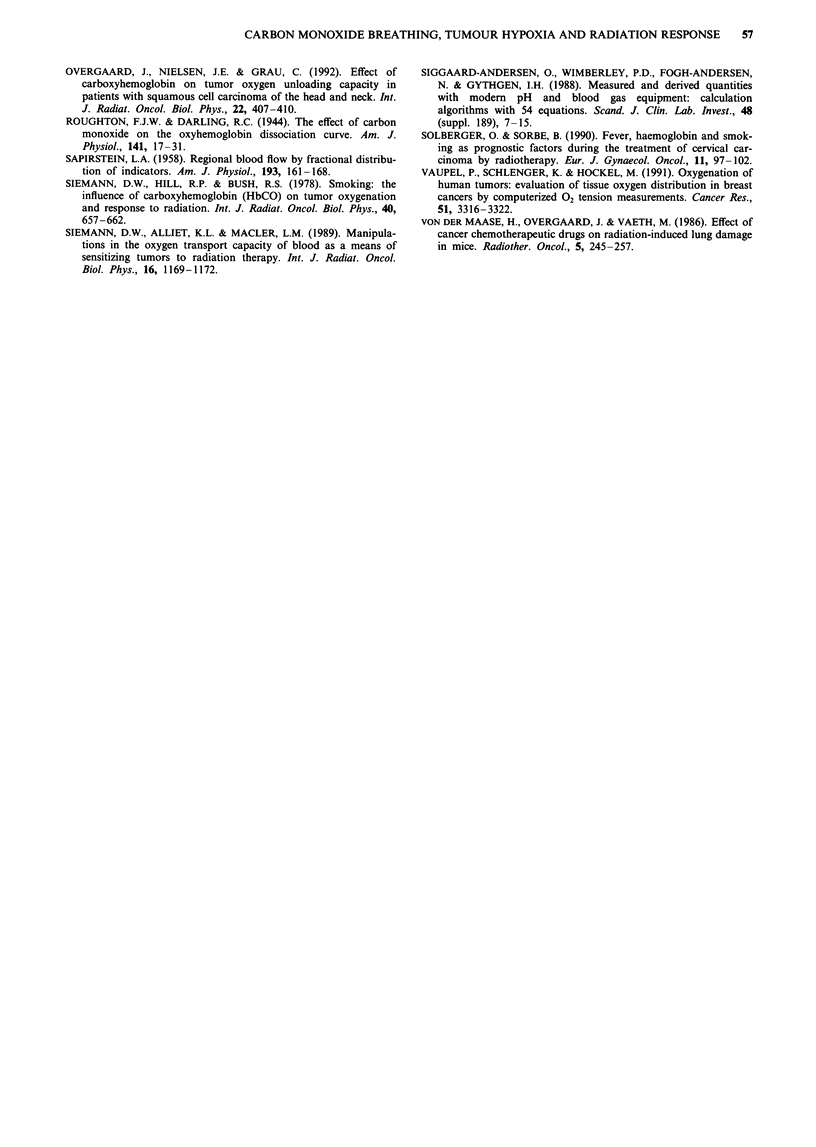# The relationship between carbon monoxide breathing, tumour oxygenation and local tumour control in the C3H mammary carcinoma in vivo.

**DOI:** 10.1038/bjc.1994.8

**Published:** 1994-01

**Authors:** C. Grau, A. A. Khalil, M. Nordsmark, M. R. Horsman, J. Overgaard

**Affiliations:** Danish Cancer Society, Department of Experimental Clinical Oncology, Aarhus.

## Abstract

The effect of acute carbon monoxide (CO) breathing on blood oxygenation and tumour hypoxia was related to the radiation response of the C3H/Tif mammary carcinoma. Blood gas analysis showed that CO breathing caused a time- and dose-dependent formation of carboxyhaemoglobin (HbCO), a significant left shift of the oxygen dissociation curve and a reduction in tumour blood perfusion. These factors all contributed to a marked drop in tumour oxygen supply. In agreement with this, tumour hypoxia was found to be significantly increased: Microelectrode PO2 measurements showed a clear relationship between CO concentration and the proportion of low PO2 measurements (< or = 5 mmHg). The fraction of clonogenic hypoxic cells increased from 8% in air-breathing animals to 13%, 18% and 54% with 75,220 and 660 p.p.m. CO respectively. The tumour hypoxia resulted in significant radiation modification. The local tumour control after single-dose and fractionated irradiation gave TCD50 enhancement ratios (relative to air-breathing controls) of 0.90, 0.85 and 0.89 for single dose and five or ten fractions given in 5 days (P < 0.005 for all values). For 15 fractions in 5 days with 6- 6- and 12 h intervals, the TCD50 was similar in CO- and air-breathing mice, presumably as a consequence of insufficient reoxygenation during the short inter-fraction intervals. It is concluded that elevated HbCO levels to increased tumour hypoxia and that the induced hypoxia has a significant impact on the local tumour control also after fractionated irradiation.


Br. J. Cancer (1994), 69, 50-57 © Macmillan Press Ltd., 1994

### The relationship between carbon monoxide breathing, tumour oxygenation and local tumour control in the C3H mammary carcinoma *in vivo*

C. Grau, A.A. Khalil, M. Nordsmark, M.R. Horsman & J. Overgaard

*Danish Cancer Society, Department of Experimental Clinical Oncology, Radiumstationen, Nørrebrogade 44, DK-8000 Aarhus C, Denmark*

**Summary** The effect of acute carbon monoxide (CO) breathing on blood oxygenation and tumour hypoxia was related to the radiation response of the C3H/Tif mammary carcinoma. Blood gas analysis showed that CO breathing caused a time- and dose-dependent formation of carboxyhaemoglobin (HbCO), a significant left shift of the oxygen dissociation curve and a reduction in tumour blood perfusion. These factors all contributed to a marked drop in tumour oxygen supply. In agreement with this, tumour hypoxia was found to be significantly increased: Microelectrode $Po_2$ measurements showed a clear relationship between CO concentration and the proportion of low $Po_2$ measurements ($\leqslant$ 5 mmHg). The fraction of clonogenic hypoxic cells increased from 8% in air-breathing animals to 13%, 18% and 54% with 75,220 and 660 p.p.m. CO respectively. The tumour hypoxia resulted in significant radiation modification. The local tumour control after single-dose and fractionated irradiation gave $TCD_{50}$ enhancement ratios (relative to air-breathing controls) of 0.90, 0.85 and 0.89 for single dose and five or ten fractions given in 5 days ($P<0.005$ for all values). For 15 fractions in 5 days with 6- 6- and 12 h intervals, the $TCD_{50}$ was similar in CO- and air-breathing mice, presumably as a consequence of insufficient reoxygenation during the short inter-fraction intervals. It is concluded that elevated HbCO levels lead to increased tumour hypoxia and that the induced hypoxia has a significant impact on the local tumour control also after fractionated irradiation.

The presence of hypoxic cells in solid tumours is well documented (Moulder & Rockwell, 1984; Gatenby *et al.*, 1988; Overgaard, 1989; Vaupel *et al.*, 1991; Horsman, 1993; Okunieff *et al.*, 1993), and tumour hypoxia is believed to be a major cause of clinical radioresistance in head and neck cancer and certain other cancer types (Dische, 1989; Overgaard, 1989; 1993). The amount of oxygen available for a given tissue depends on both the blood flow and the blood oxygenation (Hirst, 1986). The latter parameter is potentially influenced by several factors: inspired air characteristics (smoke/pollution), cardiopulmonary status, haemoglobin (Hb) amount and 'quality' and Hb-oxygen affinity (Overgaard, 1988). Apart from the registration of Hb level, details about these parameters are usually not available in clinical radiotherapy studies. A study including 115 head and neck cancer patients has recently shown that the total Hb concentration is a very crude indicator of the amount of oxygen available for tissues. Instead the concept of 'oxygen unloading capacity' has been introduced as a potentially more reliable prognostic factor (Overgaard, 1988; Overgaard *et al.*, 1992).

Tumour oxygenation is theoretically influenced by the presence of carboxyhemoglobin (HbCO), which is formed when CO binds to Hb. Even low concentrations of CO can lead to a significant level of HbCO in the blood, as the affinity of Hb for CO is about 230 times that for oxygen (Roughton & Darling, 1944). The major source of CO in the inspired air is cigarette smoke (Nordenberg *et al.*, 1990). Many patients treated with radiotherapy, especially those with head and neck cancer, are cigarette smokers (Des Rochers *et al.*, 1992; Overgaard *et al.*, 1992), and they have been found to have HbCO levels as high as 18% compared with 1-2% in non-smokers (Bunn & Forget, 1986; Nordenberg *et al.*, 1990; Overgaard *et al.*, 1992).

Previous animal studies have suggested that increased HbCO may reduce the tumour response to single-dose irradiation (Siemann *et al.*, 1978; Grau *et al.*, 1992). The aim of the present experiments was to study the relationship between blood/tumour oxygenation and tumour radiation response during CO breathing. Blood oxygenation was evaluated from blood gas analysis (HbCO, Hb, $P_{50}$), tumour perfusion from $^{86}$RbCl experiments, and the effect on tumour oxygenation was measured directly by $Po_2$ microelectrode and indirectly by radiobiological hypoxic fraction estimation.

### Material and methods

### *Mice and tumours*

The C3H mouse mammary carcinoma was grown in the feet of 10- to 14-week-old $CDF_1$/Bom (C3H/tif ♀ × DBA/2 ♂) male mice. The derivation and maintenance of the tumour system has been described in detail previously (Overgaard, 1980; Grau & Overgaard, 1988). Tumours were treated when they reached a volume of 200 mm$^3$, as determined by the formula $\pi/6 \times D_1 \times D_2 \times D_3$, where the *D*s represent the three orthogonal diameters. All experiments were performed on restrained non-anaesthetised animals.

### *Treatments and experimental techniques*

*Gassing* Specific levels of HbCO were obtained by incubating the mice in CO gas mixtures (75, 220 or 660 p.p.m. CO, ± 5%). The gases were produced and delivered by Danish Oxygen and Hydrogen Ltd. Incubation was done in a box flushed with CO at a flow rate of 5 l min$^{-1}$. The CO incubation was maintained during irradiation by moving the incubated mice to the radiation water bath, which was covered with an airtight lid and similarly flushed with CO. The HbCO levels in the treated mice were validated with blood samples on a daily basis in conjunction with each treatment session. A similar set-up was used for $^{86}$RbCl measurements. Microelectrode $Po_2$ measurements were done with the mice placed in a plastic jig, which was flushed with CO at a flow rate of 1.25 l min$^{-1}$ per jig. This flow rate was found to result in HbCO levels identical to those obtained with the incubation chamber.

*Ventilation rate* The ventilatory response to CO breathing was studied using a mouse whole-body plethysmograph (von der Maase *et al.*, 1986). The mice were placed in a 132-cm$^3$ chamber through which a constant air flow (atmospheric air or CO) was passed, and the breathing rate recorded in Hz by a built-in microphone. Results were calculated as means and s.e. of six animals in each group.

Correspondence: C. Grau
Received 10 June 1993; and in revised form 6 September 1993.

*Blood sampling* Venous blood samples (100–150 μl) were collected from the suborbital sinus and analysed for HbCO%, pH, $Po_2$, $Pco_2$, oxygen saturation and total Hb% on an Acid Base Laboratory ABL300 (Radiometer, Copenhagen) connected to an OSM3 haemoximeter (Radiometer, Copenhagen) calibrated for mouse blood. The $P_{50}$ was calculated automatically from these values (Siggaard-Andersen *et al.*, 1988). This value is defined as the oxygen partial pressure at which Hb is 50% saturated with oxygen at normal pH, and it thus describes the position of the Hb–oxygen dissociation curve. A fraction of the blood samples was analysed for the content of 2,3-diphosphoglycerate (2,3-DPG). This was done by a commercial kit (Sigma, St Louis, MO, USA). In brief, the breakdown of 2,3-DPG to glyceraldehyde 3-phosphate requires simultaneous formation of NAD, which can be quantitated spectrophotometrically. The test was done on protein-free supernatant, and the values calculated either as whole-blood concentration (mmol $l^{-1}$) or as a percentage of the haemoglobin content, the latter parameter calculated by dividing the whole-blood 2,3-DPG by the Hb content.

*Intratumoral* $Po_2$ *measurement* The intratumoral oxygen tension was measured using a fine-needle polarographic electrode (Eppendorf, Hamburg, Germany), as described previously (Horsman *et al.*, 1993). In brief, $Po_2$ measurements were conducted with mice restrained in lucite jigs with the tumour-bearing foot exposed and loosely taped in such a way that the normal blood supply was not impaired. The needle was inserted up to a depth of 1 mm into the tumour, and automatically moved through the tumour in forward steps of 0.7 mm followed by a rapid retraction of 0.3 mm. Three to six electrode tracks were used in each tumour, yielding a total of 45–90 measurements per tumour. The relative frequency of the $Po_2$ was automatically calculated and displayed as a histogram. For the present purpose, the data were expressed as the percentage of measurements with a $Po_2$ value less than or equal to 5 mmHg.

*Tumour blood perfusion measurement* Tumour blood perfusion was measured by the $^{86}$RbCl extraction technique (Sapirstein, 1958). The practical application of this technique in our set-up has been described in detail previously (Horsman *et al.*, 1989; Grau *et al.*, 1992). Briefly, a volume of 0.1 ml of $^{86}$RbCl was injected intravenously into each mouse. After 2 min the mice were sacrificed and the tumour-bearing leg was clamped. Tumours were excised for counting immediately after sacrifice. Determinations of radioactivity were made on a gamma-counter, and the radioactive counts were expressed as percentage injected per g of tumour.

*Irradiation* Local irradiation was given with 250-kV X-rays (2.3 Gy $min^{-1}$, 3.1 mm Cu HVL, 10 mA) to mice with the tumour-bearing foot immersed in a water bath (25°C) to secure homogeneous dose distribution. Fractionated irradiation was given with an interval between multiple daily fractions of 6 h, and an overall treatment time of 4–4½ days. Animals receiving X-rays under hypoxic conditions had the tumour-bearing leg clamped 5 min before and during the period of irradiation. Clamping was achieved by constriction of the blood flow using a rubber tube tightened around the leg (Grau *et al.*, 1992).

### *End-points and statistics*

*Local tumour control assay* The effect of graded doses of radiation was evaluated as the dose required to produce local tumour control in 50% of the treated animals ($TCD_{50}$). Tumour control was defined as complete absence of macroscopic relapse within 90 days. Each single experiment included 6–9 dose points each with 6–12 mice. Less than 5% of the animals were censored as a result of death, amputation or distant metastases. Data were analysed by logit analysis (Grau & Overgaard, 1988). The enhancement ratio (ER) was calculated as the ratio of $TCD_{50}$ values obtained under normal air-breathing and CO-breathing conditions. An ER significantly lower than 1 thus reflected radiation protection.

*Hypoxic fraction* The proportion of radiobiologically hypoxic cells (HF) was estimated from the tumour control data obtained for mice breathing CO at 0, 75, 220, or 660 p.p.m. together with data for the clamped tumours. One-step direct estimation was performed using a modification of the 'αβest' computer program, with fixed α- and β-values of 0.53 $Gy^{-1}$ and 0.087 $Gy^{-2}$ respectively (Bentzen & Grau, 1991).

*Statistical methods* The experimental data were calculated as means and 95% confidence limits using normal distribution unless otherwise stated in the text. Student's *t*-test with a significance level of 5% was used in all analyses. Univariate linear regression analysis (SPSS/PC + V4.01) was used to test for correlation. The estimation of HF from tumour control data and its confidence intervals were calculated from the 'αβest' computer program, as described by Bentzen & Grau (1991).

### Results

### *Breathing rate*

Figure 1 shows the ventilation rate in CO- and air-breathing mice. The initial breathing rate was about 270 $min^{-1}$, but when the mice became accustomed to the chamber this value dropped to about 200 $min^{-1}$. There was no difference between air- and CO-breathing animals. This HbCO level at 90 min was 25%.

### *Blood oxygenation*

The effect of acute and long-term exposure to various levels of CO on HbCO and $P_{50}$ as a function of breathing time is shown in Figure 2. Thirty non-tumour-bearing mice were 'incubated' for each concentration at time zero. Blood samples were taken from three mice at each time point. For practical and ethical reasons each mouse was measured only once from each orbital sinus during the experiment. The experiment was repeated once for each CO concentration. One data point thus represents the mean and 95% confidence interval of six different mice (and not the same mouse followed consecutively). The results show that air-breathing mice had an HbCO level of about 2%. After exposure to CO

**Figure 1** The ventilation rate determined by whole-body plethysmography in mice breathing either air (○) or CO 220 p.p.m. (●). Points are mean and 95% confidence limits of six individual measurements.

**Figure 2** Blood oxygenation parameters in non-tumour-bearing mice as a function of breathing time in CO 75 p.p.m. (○), 220 p.p.m (●), or 660 p.p.m. (▽). **a**, Carboxyhaemoglobin (HbCO). **b**, *P*50. Shaded areas are 95% confidence intervals of the control value. Error bars are 95% confidence intervals.

**Figure 3** The correlation between blood carboxyhaemoglobin (HbCO) level and **a**, $P_{50}$ and **b**, total haemoglobin (○) or effective haemoglobin (●). Points are individual measurements. Line drawn by linear regression.

the HbCO level rapidly increased, and reached dose-dependent maximal values of approximately 10% (75 p.p.m.), 25% (220 p.p.m.) and 45% (660 p.p.m.) within the first 24 h.

The breathing of CO caused an increase in oxygen affinity and a reduction in $P_{50}$ within minutes. The $P_{50}$ describes the position of the oxygen dissociation curve, and for normal air-breathing mice it was 38 mmHg (Figure 2b). The maximal reduction in $P_{50}$ with time was about 25% for CO 75 p.p.m., 35% for 220 p.p.m. and 50% for 660 p.p.m. The strong inverse relationship between the increase in HbCO and the drop in $P_{50}$, the so-called Haldane effect, is shown in Figure 3a, in which the individual measurements of these parameters are plotted against each other. There was no change in total Hb as a function of HbCO level or breathing times up to 4 days. In fact, in another experiment the Hb content was unchanged during a 3-week chronic CO-breathing period (data not shown). Although the total Hb content remained constant, the amount of Hb available for oxygen transportation was significantly reduced by CO breathing. The so-called effective Hb, defined as total Hb – (HbCO + MetHb), is shown as a function of HbCO level in Figure 3b. The MetHb concentration was independent of all manipulations, and was 0.5% in this mouse strain.

One of the factors that strongly influences the $P_{50}$ is the 2,3-DPG concentration. This parameter was obtained from approximately half of the animals included in the blood gas analysis. In contrast to what was expected, 2,3-DPG did not increase as a response to the hypoxic stimulus from CO breathing, but rather showed a slight decrease with longer breathing time. This was true for both haemoglobin 2,3-DPG (Figure 4) and whole-blood 2,3-DPG (data not shown). There was no correlation between the CO concentrations and the 2,3-DPG response. Similarly, there was no correlation between the 2,3-DPG level and either HbCO, effective Hb or $P_{50}$ level.

In the tumour experiments a pretreatment breathing time

**Figure 4** The 2,3-DPG (as a percentage of HB content) as a function of breathing time in CO 75 p.p.m. (○), 220 p.p.m. (●) or 660 p.p.m. (▽). Shaded areas are 95% confidence intervals of the control value. Error bars are 95% confidence intervals.

of 45–60 min was used, by which time the blood oxygenation parameters had stabilised at a dose-dependent level. The HbCO or $P_{50}$ values cited below refer to those values actually measured in conjunction with each investigation or treatment (from simultaneously gassed controls or in treated animals). The values are thus *not* transposed from the blood gas experiments.

### *Tumour blood perfusion*

Tumour blood perfusion was measured by the $^{86}RbCl$ extraction technique (Figure 5). The mice were breathing CO for

**Figure 5** The effect of HbCO on tumour perfusion measured by the $^{86}$RbCl technique. Line drawn by linear regression. Shaded area represents perfusion in air-breathing controls (95% confidence intervals).

45–60 min prior to $^{86}$RbCl injection. There was a significant correlation between the HbCO level and the decrease in tumour perfusion ($r^2 = 0.98$, $P < 0.001$). The reductions in blood perfusion were 12% (NS), 45% ($P < 0.05$) and 82% ($P < 0.01$) for average HbCO levels of 13%, 25% and 51% respectively.

The combined effects of changes in effective Hb and tumour blood perfusion on the oxygen supply to tumours are illustrated in Figure 6. All values are expressed as percentage of the values obtained in air-breathing controls. The perfusion effects are represented with the regression line from Figure 5. The combined effect was calculated as the product of the reduction in effective Hb and the estimated perfusion reduction for animals at the actual HbCO level for each mouse. It is seen that the negative impact of perfusion is considerably greater than the reduction in effective Hb. When combined, the amount of oxygen supplied to the tumour is decreased down to 15% of the control value for the highest HbCO level. Within the clinically relevant range, the reduction is about 30–40%.

**Figure 6** The effect of HbCO on tumour oxygen supply. All values are relative to air-breathing controls. □, total Hb; ○, effective Hb; - - -, blood perfusion; ●, oxygen supply, calculated as the ratio of effective Hb to perfusion for air- and CO-breathing animals.

*Tumour oxygenation*

The percentage of intratumoral microelectrode $Po_2$ measurements with values less than or equal to 5 mmHg as a function of the blood HbCO level is plotted in Figure 7a. Mice were breathing atmospheric air or CO for 45–60 min before and continuously during measurement. In air-breathing mice 35% of measurements contained $Po_2$ values less than or equal to 5 mmHg. This percentage rapidly increased with increasing levels of HbCO. The value at CO 660 p.p.m. (81%) was not significantly different from that obtained in clamped tumours (97%). The proportion of clonogenic hypoxic cells (HF) was measured radiobiologically using the clamped local tumour control technique. The tumour control data obtained for mice breathing CO at 0, 75, 220 or 660 p.p.m. for 45 min before and during local radiotherapy were analysed together with the data for clamped irradiation. The HF values obtained at different HbCO levels are shown in Figure 7b, and listed in Table I. The HF increased from 8% in air-breathing animals to 13% (75 p.p.m.), 18% (220 p.p.m.) and 54% (660 p.p.m.). The correlation was highly significant ($r^2 = 0.97$, $P < 0.05$).

The correlation between HbCO and HF was significant ($r^2 = 0.97$, $P < 0.05$) and similar to that observed for the $Po_2$.

*Radiation response*

The effect of CO breathing on the radiation-induced local tumour control was studied with single dose and fractionated irradiation. The $TCD_{50}$ data are summarized in Table I. Figure 8a shows the significant correlation between the HbCO level and the obtained $TCD_{50}$ for single-dose irradiation ($r^2 = 0.98$, $P < 0.05$). The responses of clamped tumours are shown for comparison (shaded area). The negative effect of HbCO on radiation response is plotted in a more clinically relevant way in Figure 8b. This plot illustrates the loss of

**Figure 7** The effect of HbCO on **a**, percentage of intratumoral $Po_2$ values less than or equal to 5 mmHg and **b**, radiobiological hypoxic fraction of clonogenic cells, determined by direct analysis of tumour control data for ambient and clamped tumours respectively. Lines drawn by linear regression. Shaded areas represent values from air-breathing controls (95% confidence intervals).

**Table I** Effect of carbon monoxide breathing on the radiation-induced local tumour control and proportion of radiobiologically hypoxic cells of a C3H mammary carcinoma *in vivo*

| *Rad.* (n)[a] | *Breathing condition* | *HbCO (%)*[b] | *$TCD_{50}$ (Gy)* | *Enhancement ratio*[c] | *Hypoxic fraction (%)* |
|---|---|---|---|---|---|
| 1 | Air | 1-2 | 54 (52-56) | – | 8 (5-10) |
| 1 | CO 75 p.p.m. | 7-9 | 57 (54-61) | 0.94 (0.89-0.99)[f] | 13 (7-18) |
| 1 | CO 220 p.p.m. | 20-23 | 60 (56-63) | 0.90 (0.85-0.95)[f] | 18 (10-26)[f] |
| 1 | CO 660 p.p.m. | 27-31 | 68 (64-71) | 0.80 (0.76-0.84)[f] | 54 (32-76)[f] |
| 1 | Clamped | 1-2 | 71 (69-73) | – | 100 (68-132)[f] |
| 5 | Air | 1-2 | 61 (59-63) | – | 1.5 (0.4-2.6) |
| 5 | CO 220 p.p.m. acute[d] | 16-18 | 71 (64-79) | 0.85 (0.78-0.93)[f] | 3.0 (0.7-5.4) |
| 5 | CO 220 p.p.m. chron.[e] | 10-14 | 67 (61-73) | 0.91 (0.84-0.98)[f] | 2.1 (0.5-3.6) |
| 5 | Clamped | 1-2 | 113 (105-120) | – | 100 (43-158)[f] |
| 10 | Air | 1-2 | 87 (83-91) | – | 13 (6-20) |
| 10 | CO 220 p.p.m. | 15 x 19 | 97 (94-101) | 0.89 (0.85-0.94)[f] | 26 (9-43) |
| 10 | Clamped | 1-2 | 119 (111-127) | – | 100 (23-177)[f] |
| 15 | Air | 1-2 | 97 (92-103) | – | 40 (16-65) |
| 15 | CO 220 p.p.m. | 14-19 | 98 (92-105) | 0.99 (0.93-1.06) | 37 (14-60) |
| 15 | | 1-2 | 115 (105-126) | – | 100 (33-167)[f] |

[a]Radiation given in *n* fractions in a total overall treatment time of 5 days. [b]Carboxyhaemoglobin (HbCO;range of measured values pre and post-irradiation). [c]Defined as $TCD_{50}$ for air-breathing animals relative to CO-treating animals. [d]Exposed to CO only 45 min before the during treatment. [e]Exposed chronically to a CO-containing environment from tumour inoculation until the end of treatment. [e]Exposed chronically to a CO-containing environment from tumour inoculation until the end of treatment. [f]$P<0.05$ (compared with air-breathing control). Numbers in brackets are 95% confidence limits.

**Figure 8** The effect of HbCO on **a**, local tumour control ($TCD_{50}$) and **b**, tumour control rate at 60 Gy single-dose irradiation. Shaded areas represent response in clamped or air-breathing controls (95% confidence intervals). Error bars are 95% confidence intervals.

**Figure 9** Local tumour control for irradiation (one, five, ten or 15 fractions) in air-breathing (○), acute 220 p.p.m. CO-breathing (●) or chronic 110 p.p.m. CO-breathing (▽) mice. The dotted lines indicate the response of clamped tumours. Error bars are 95% confidence intervals.

tumour control for a fixed radiation dose of 60 Gy. The 80% local tumour control rate in air-breathing mice declined to about 50% within the clinically relevant HbCO range. The correlation was significant ($r^2 = 0.99$, $P<0.01$).

The influence of HbCO on local tumour control was further investigated in a series of fractionated radiation experiments. An overall treatment time of 5 days was used for all schedules, Figure 9 shows the local tumour control as a function of the total radiation dose. For comparison, the responses of clamped tumours are indicated with dashed lines. There was a small, but highly significant increase in $TCD_{50}$ when the radiation was given in one, five or ten fractions. This effect could not be found for 15 fractions, in which case the two dose–response curves overlapped. The calculated $TCD_{50}$ and the corresponding enhancement ratios are listed in Table I. For one, five and ten fractions, the radiation ERs were 0.90, 0.85 and 0.89 respectively, which was highly significant ($P<0.005$ for all values). Finally, chronic smoking was simulated in a single experiment. Mice maintained from tumour implantation until the end of the five-fraction radiation treatment – a period of about 21 days – in a 110 p.p.m. CO environment (HbCO 10–14%) had a $TCD_{50}$ that was not significantly different from that observed for mice that were only acutely exposed to 220 p.p.m. CO during treatment (Figure 9, bottom left). The $TCD_{50}$ was significantly higher than for air-breathing controls ($P < 0.05$). The Hb levels of these chronic breathers were identical to those of untreated controls (data not shown).

### Discussion

This study has demonstrated that CO breathing causes a significant decrease in tumour oxygen supply, which in turn leads to severe tumour hypoxia and a reduction in radiation response. It is established that the tumour oxygen supply is impaired by three factors: reduction in effective Hb from HbCO formation, decrease in $P_{50}$ and reduction in tumour blood perfusion. The combined effect results in at least 30–40% reduction in oxygen supply to the tumour within the clinical HbCO range, and up to 85% for high CO concentrations.

Our data show a dose-dependent reduction in $P_{50}$ down to about 50% of the normal value. Such increase in blood oxygen affinity will decrease the oxygen utilisation depending on the tissue $Po_2$ (Overgaard *et al.*, 1992). It is not yet possible to quantify exactly these changes in mouse blood in the same way as has been done in humans (Overgaard *et al.*, 1992). However, an impression of the relative magnitude of the Haldane effect can be obtained if the tissue $Po_2$ is kept constant. Then the effect of a reduction in $P_{50}$ would be of the same magnitude as the perfusion effect, i.e. up to 80% reduction at the highest HbCO. A reliable estimate of the tumour $Po_2$ can be obtained from the microelectrode measurements. In the present experiments the mean $Po_2$ decreased linearly from 14 mmHg (air) to 3 mmHg (CO 660 p.p.m.). Such a drop with increasing tumour hypoxia will facilitate oxygen release because of the increased concentration gradient, and thereby to some extent counteract the $P_{50}$ effect.

A reduction in $P_{50}$ has also been observed for several therapeutic agents. These agents include the substituted benzaldehyde BW12C, which preferentially binds to oxyhaemoglobin and thereby increases the affinity of Hb for oxygen (Beddell *et al.*, 1984; Horsman & Overgaard, 1992). Several experimental studies have shown a significant increase in tumour hypoxia and a reduction in radiation response when mice are treated with BW12C (Adams *et al.*, 1986; Adams, 1989; Honess *et al.*, 1989). However, recent studies have suggested that the observed radiation modification may be more related to BW12C-induced blood flow reductions than to the change in $P_{50}$ (Honess *et al.*, 1991, Horsman & Overgaard, 1992). A similar property was observed for CO breathing, when perfusion reductions up to 80% were seen at the highest CO concentration. Previous studies from our laboratory with the C3H mouse mammary carcinoma and the vasoactive agent hydralazine showed that at least a 50% decrease in perfusion is required to see any effect on radiation response and full radiobiological hypoxia is only seen after a 90% reduction in perfusion (Horsman *et al.*, 1989). The Hb affinity for oxygen can also be *decreased* in order to improve tumour radiosensitivity. This has been shown experimentally with antilipidaemic agents (Hirst & Wood, 1987; 1989; Hirst *et al.*, 1987) and a combination of inosine, puruvate and phosphate (2,3-DPG precursors) (Siemann *et al.*, 1989). It is not known to what extent blood perfusion changes are involved in the mechanisms of action of these agents.

2,3-DPG is one of the most important allosteric factors controlling the position of the oxygen dissociation curve (Bunn & Forget, 1986). In conditions characterised by hypoxia (chronic lung disease, cardiac insufficiency, anaemia), the level of 2,3-DPG is increased, thereby making the oxygenated Hb more readily available to the tissues by an increase in the $P_{50}$. This effect could not be seen in the present study. A possible reason is that the hypoxic stimulus from HbCO is not sufficient to trigger a sufficient biochemical response.

The chronic effects of smoking have generally been believed to include a compensatory polycythaemia. However, the magnitude of such polycythaemia in persons without chronic lung disease is very small. In a large study of more than 4000 persons the Hb level in smokers was less than 3% above that of non-smokers (Nordenberg *et al.*, 1990), and in head and neck cancer patients no increase in Hb as a function of HbCO level has been found (Overgaard *et al.*, 1992). In the present mouse material we found no increase in Hb during the 3 weeks of chronic 'incubation' at 10% HbCO. So it is unlikely that chronic CO breathing/smoking leads to any significant adaptation, which is also shown by the data in Figure 9. The present set-up with acute CO breathing therefore seems to be a reasonable simulation of the clinical situation.

The local tumour control studies showed that even within the clinically relevant HbCO range the radiation response was significantly reduced for single-dose and fractionated irradiation with five or ten fractions. Similar or greater radioprotection has been found in the SCCVII tumour using the *in vivo* excision assay (C. Grau, unpublished observations, 1993). Mice with a HbCO of 10–13% had isoeffect ERs between 0.76 and 0.83 for one, four, eight or 12 fractions within 4 days. In the KHT tumour, Siemann *et al.* (1978) similarly found significantly increased tumour cell survival when tumours were given daily fractionation with 5 Gy during either acute or chronic exposure to CO giving a 10% HbCO level.

The only schedule in the present set-up where CO breathing did not decrease the radiation response was when 15 fractions were delivered as three daily fractions. A possible explanation of this finding may be that reoxygenation in such a hyperfractionated setting is not complete between fractions, and the contribution of 'CO-induced hypoxia' is therefore less important. There are some data to support this hypothesis. Using the previously published local tumour control data for clamped tumours (Bentzen & Grau, 1991), it was possible to calculate the 'effective' hypoxic fractions for the different fractionation schedules similar to what was done for the single-dose data. For five fractions (i.e. 24 h interval) the effective hypoxic fraction was significantly lower than that for untreated tumours (1.5% *vs* 8%), indicating that reoxygenation between fractions was very efficient (Table I and Figure 10). The hypoxic fraction was increased by a factor of 2 by CO breathing. For twice-daily fractionation (i.e. 6 and 18 h intervals) the hypoxic fraction was 13% in aerobic tumours, indicating that reoxygenation was almost complete. Again, CO breathing caused a doubling of the hypoxic fraction. For three daily fractions (i.e. 6- 6- and 12 h

**Figure 10** The 'effective' hypoxic fraction during different fractionation schedules in air-breathing (□) or CO-breathing (■) mice. Error bars are 95% confidence intervals.

intervals), however, the effective hypoxic fraction increased to 40%, possibly as a result of insufficient reoxygenation. In this situation CO breathing did not further increase the hypoxic fraction.

The clinical data on the influence of smoking on radiation response are remarkably sparse. A recent prospective study has shown that the survival of smokers undergoing radiotherapy for head and neck cancer is significantly reduced compared with non-smokers of the same clinical stage (Browman *et al.*, 1993). The 2-year disease-free survival rate was 66% in non-smokers compared with 39% in the patients who continued to smoke during treatment ($P = 0.005$). The conclusion of the study was that patients should be advised to stop smoking during therapy. Similar suggestions have been made retrospectively in carcinoma of the uterine cervix (Kucera *et al.*, 1987), but in another study in the same patient category smoking was found to be a non-significant prognostic factor (Solberger & Sorbe, 1990). Protection of the normal tissue by CO breathing may influence the therapeutic importance of the present findings. However, in a recent study (Browman *et al.*, 1993), no difference in the incidence and severity of stomatitis and skin toxicity between smokers and abstainers was found. Apart from these studies there exist (at least to our knowledge) no good data on this topic.

In conclusion, the present data have shown that elevated HbCO levels can lead to increased tumour hypoxia as a result of changes in blood oxygenation and tumour blood perfusion, and that the induced hypoxia has a significant impact on the local tumour control after both single-dose and fractionated irradiation.

The authors wish to thank Ms Marianne H. Simonsen, Ms Mette Andersen, Ms Alice Baden and Ms Inger Marie Johansen for excellent technical assistance. This study was supported by a grant from the Danish Cancer Society.

### References

ADAMS, G.E., BARNES, D.W.H., DUBOULAY, C., LOUTIT, J.F., COLE, S., SHELDON, P.W., STRATFORD, I.J., VAN DEN AARDWEG, G.J.M.J., HOPEWELL, J.W., WHITE, R.D., KNEEN, G., NETHERSELL, A.B.W. & EDWARDS, J.C. (1986). Induction of hypoxia in normal and malignant tissues by changing the oxygen affinity of haemoglobin – implications for therapy. *Int. J. Radiat. Oncol. Biol. Phys.*, **12**, 1299–1302.

ADAMS, G.E. (1989). Induction of severe tumor hypoxia by modifiers of the oxygen affinity of hemoglobin. *Int. J. Radiat. Oncol. Biol. Phys.*, **16**, 1179–1182.

BEDDELL, C.R., GOODFORD, P.J., KNEEN, G., WHITE, R.D., WILKINSON, S. & WOOTON, R. (1984). Substituted benzaldehydes designed to increase the oxygen affinity of human haemoglobin and inhibit the sickling of sickle erythrocytes. *Br. J. Pharmacol.*, **82**, 397–407.

BENTZEN, S.M. & GRAU, C. (1991). Direct estimation of the fraction of hypoxic cells from tumor-control data obtained under aerobic and clamped conditions. *Int. J. Radiat. Biol.*, **59**, 1435–1440.

BROWMAN, G.P., WONG, G., HODSON, I., SATHYA, J., RUSSELL, R., MCALPINE, L., SKINGLEY, P. & LEVINE, M.N. (1993). Influence of cigarette smoking on the efficacy of radiation therapy in head and neck cancer. *N. Engl. J. Med.*, **328**, 159–163.

BUNN, H.F. & FORGET, B.G. (1986). *Hemoglobin: Molecular, Genetic and Clinical Aspects*. W.B. Saunders: Philadelphia.

DES ROCHERS, C., DISCHE, S. & SAUNDERS, M.I. (1992). The problem of cigarette smoking in radiotherapy for cancer in the head and neck. *Clin. Oncol.*, **4**, 214–216.

DISCHE, S. (1989). The clinical consequences of the oxygen effects. In *The Biological Basis of Radiotherapy*. Steel, G.G., Adams, G.E. & Horwich, A. (eds) p. 135. Elsevier Science Publishers: Amsterdam.

GATENBY, R.A., KESSLER, H.B., ROSENBLUM, J.S., COIA, L.R., MOLDOFSKY, P.J., HARTZ, W.H. & BRODER, G.J. (1988). Oxygen distribution in squamous cell carcinoma metastases and its relationship to outcome of radiation therapy. *Int. J. Radiat. Oncol. Biol. Phys.*, **14**, 831–838.

GRAU, C. & OVERGAARD, J. (1988). Effect of cancer chemotherapy on the hypoxic fraction in a solid tumor measured using a local tumor control assay. *Radiother. Oncol.*, **13**, 301–309.

GRAU, C., HORSMAN, M.R. & OVERGAARD, J. (1992). Influence of carboxyhemoglobin level on tumor growth, blood flow, and radiation response in an experimental model. *Int. J. Radiat. Oncol. Biol. Phys.*, **22**, 421–424.

HIRST, D.G. (1986). Oxygen delivery to tumors. *Int. J. Radiat. Oncol. Biol. Phys.*, **12**, 1271–1277.

HIRST, D.G. & WOOD, P.J. (1987). The influence of haemoglobin affinity for oxygen on tumour radiosensitivity. *Br. J. Cancer*, **55**, 487–491.

HIRST, D.G. & WOOD, P.J. (1989). Chlorophenoxy acetic acid derivatives as hemoglobin modifiers and tumor radiosensitizers. *Int. J. Radiat. Oncol. Biol. Phys.*, **16**, 1183–1186.

HIRST, D.G., WOOD, P.J. & SCHWARTZ, H.C. (1987). The modification of hemoglobin affinity for oxygen and tumor radiosensitivity by antilipidemic drugs. *Radiat. Res.*, **112**, 164–172.

HONESS, D.J., WHITE, R.D., NETHERSELL, A.B.W. & BLEEHEN, N.M. (1989). Effects of manipulation of oxyhaemoglobin status by BW12C on tumor thermosensitivity and on blood flow in tumor and normal tissues in mice. *Int. J. Radiat. Oncol. Biol. Phys.*, **16**, 1187–1190.

HONESS, D.J., HU, D.E. & BLEEHEN, N.M. (1991). BW12C: effects on tumour hypoxia, tumour radiosensitivity and relative tumour and normal tissue perfusion in C3H mice. *Br. J. Cancer*, **64**, 715–722.

HORSMAN, M.R. & OVERGAARD, J. (1992). BW12C-induced changes in haemoglobin–oxygen affinity in mice and its influence on the radiation response of a C3H mouse mammary carcinoma. *Radiother. Oncol.*, **25**, 43–48.

HORSMAN, M.R. (1993). Hypoxia in tumors: its relevance, identification and modification. In *Current Topics in Clinical Radiobiology of Tumours*, Beck-Bornholdt, H.P. (ed.) (in press).

HORSMAN, M.R., CHRISTENSEN, K.L. & OVERGAARD, J. (1989). Hydralazine-induced enhancement of hyperthermic damage in a C3H mammary carcinoma in vivo. *Int. J. Hyperthermia*, **5**, 123–136.

HORSMAN, M.R., KHALIL, A.A., NORDSMARK, M., GRAU, C. & OVERGAARD, J. (1993). Relationship between radiobiological hypoxia and direct estimates of tumour oxgenation in a mouse tumour model. *Radiother. Oncol.*, **28**, 69–71.

KUCERA, H., ENZELSBERGER, H., EPPEL, W. & WEGHAUPT, K. (1987). The influence of nicotine abuse and diabetes mellitus on the results of primary irradiation in the treatment of carcinoma of the cervix. *Cancer*, **60**, 1–4.

MOULDER, J.E. & ROCKWELL, S. (1984). Hypoxic fractions of solid tumours: experimental techniques, methods of analysis, and a survey of existing data. *Int. J. Radiat. Oncol. Biol. Phys.*, **10**, 695–712.

NORDENBERG, D., YIP, R. & BINKIN, N.J. (1990). The effect of cigarette smoking on hemoglobin levels and anemia screening. *JAMA*, **264**, 1556–1559.

OKUNIEFF, P., HOCKEL, M., DUNPHY, E.P., SCHLENGER, K., KNOOP, C. & VAUPEL, P. (1993). Oxygen tension distributions are sufficient to explain the local response of human breast tumors treated with radiation alone. *Int. J. Radiat. Oncol. Biol. Phys.*, **26**, 631–636.

OVERGAARD, J. (1980). Effect of misonidazole and hyperthermia on the radiosensitivity of a C3H mouse mammary carcinoma and its surrounding normal tissue. *Br. J. Cancer*, **41**, 10–21.

OVERGAARD, J. (1988). The influence of haemoglobin concentration on the response to radiotherapy. *Scand. J. Clin. Lab. Invest.*, **48** (suppl. 189), 49–53.

OVERGAARD, J. (1989). Sensitization of hypoxic tumour cells – clinical experience. *Int. J. Radiat. Biol.*, **56**, 801–811.

OVERGAARD, J. (1993). Advances in clinical applications of radiobiology: phase III studies of radiosensitizers and novel fractionation schedules. In *Head and Neck Cancer*, Vol. III. Elsevier Science Publishers: Amsterdam.

OVERGAARD, J., NIELSEN, J.E. & GRAU, C. (1992). Effect of carboxyhemoglobin on tumor oxygen unloading capacity in patients with squamous cell carcinoma of the head and neck. *Int. J. Radiat. Oncol. Biol. Phys.*, **22**, 407–410.

ROUGHTON, F.J.W. & DARLING, R.C. (1944). The effect of carbon monoxide on the oxyhemoglobin dissociation curve. *Am. J. Physiol.*, **141**, 17–31.

SAPIRSTEIN, L.A. (1958). Regional blood flow by fractional distribution of indicators. *Am. J. Physiol.*, **193**, 161–168.

SIEMANN, D.W., HILL, R.P. & BUSH, R.S. (1978). Smoking: the influence of carboxyhemoglobin (HbCO) on tumor oxygenation and response to radiation. *Int. J. Radiat. Oncol. Biol. Phys.*, **40**, 657–662.

SIEMANN, D.W., ALLIET, K.L. & MACLER, L.M. (1989). Manipulations in the oxygen transport capacity of blood as a means of sensitizing tumors to radiation therapy. *Int. J. Radiat. Oncol. Biol. Phys.*, **16**, 1169–1172.

SIGGAARD-ANDERSEN, O., WIMBERLEY, P.D., FOGH-ANDERSEN, N. & GYTHGEN, I.H. (1988). Measured and derived quantities with modern pH and blood gas equipment: calculation algorithms with 54 equations. *Scand. J. Clin. Lab. Invest.*, **48** (suppl. 189), 7–15.

SOLBERGER, O. & SORBE, B. (1990). Fever, haemoglobin and smoking as prognostic factors during the treatment of cervical carcinoma by radiotherapy. *Eur. J. Gynaecol. Oncol.*, **11**, 97–102.

VAUPEL, P., SCHLENGER, K. & HOCKEL, M. (1991). Oxygenation of human tumors: evaluation of tissue oxygen distribution in breast cancers by computerized $O_2$ tension measurements. *Cancer Res.*, **51**, 3316–3322.

VON DER MAASE, H., OVERGAARD, J. & VAETH, M. (1986). Effect of cancer chemotherapeutic drugs on radiation-induced lung damage in mice. *Radiother. Oncol.*, **5**, 245–257.